# Protection of Piglets with Maternally Derived Antibodies from Sows Inoculated with an Attenuated Live Marker Classical Swine Fever Vaccine (Flc-LOM-BE^rns^)

**DOI:** 10.3390/pathogens9080608

**Published:** 2020-07-27

**Authors:** SeEun Choe, Jihye Shin, Ki-Sun Kim, Sok Song, Ra Mi Cha, Byung-Il Jung, Bang-Hun Hyun, Bong-Kyun Park, Dong-Jun An

**Affiliations:** 1 Virus Disease Division, Animal and Plant Quarantine Agency, Gimchen, Gyeongbuk-do 39660, Korea; ivvi59@korea.kr (S.C.); shinji227@korea.kr (J.S.); kisunkim@korea.kr (K.-S.K.); ssoboro9@korea.kr (S.S.); rami.cha01@korea.kr (R.M.C.); hyunbh@korea.kr (B.-H.H.); parkx026@korea.kr (B.-K.P.); 2 Korea Pork Producers Association, Seocho-gu, Seoul 06643, Korea; exksa001@daum.net; 3 College of Veterinary Medicine, Seoul University, Gwanak-ro, Gwanak-gu, Seoul 08826, Korea

**Keywords:** CSFV, DIVA, Flc-LOM-BE^rns^ vaccine, MDA

## Abstract

Here, we investigated the protective efficacy provided by passive immunity induced by a classical swine fever (Flc-LOM-BE^rns^) vaccine with the newly developed DIVA (Differentiating Infected from Vaccinated Animals) function. Ten pigs (aged 40–60 days) with maternally derived antibodies (MDAs) obtained from sows inoculated with the Flc-LOM-BE^rns^ vaccine were challenged with virulent classical swine fever virus (CSFV). Pigs with an MDA titer of 6 log_2_ induced by the Flc-LOM-BE^rns^ vaccine were fully protected against virulent CSFV challenge but not the pigs with an MDA titer under 5 log_2_. In addition, Flc-LOM-BE^rns^ vaccine-derived MDAs successfully differentiated vaccinated pigs by bovine viral diarrhea virus (BVDV) E^rns^/CSFV E^rns^ antibody detection, functioning as a DIVA.

## 1. Introduction

Classical swine fever virus (CSFV) is a member of the genus *Pestivirus* (family, *Flaviviridae*) [1,2,3]. CSFV harbors a single-stranded positive RNA genome comprising approximately 12,300 nucleotides. CSFV is classified into three different genotypes, which in turn comprise three or four sub-genotypes [1,2,3]. CSFV is a highly contagious, multi-systemic, hemorrhagic disease that is fatal to swine (*Sus scrofa*); it infects both breeding pigs and wild boars [1]. The development of DIVA (Differentiating Infected from Vaccinated Animals) vaccines, which make it possible to distinguishing vaccinated animals from infected animals, has drawn great attention [2]. A CSF live marker vaccine (Flc-LOM-BE^rns^) that functions as a DIVA was generated by inserting the Erns gene from bovine viral diarrhea virus (BVDV) into an infectious clone (Flc-LOM) derived from the LOM strain, which was used as a CSF vaccine in South Korea [4]. Pigs inoculated with Flc-LOM-BE^rns^ vaccine induced antibody against BVDV E^rns^ but not CSFV E^rns^. After virulent CSFV challenge, vaccine immunized pigs would have both antibodies against BVDV E^rns^ and CSFV E^rns^. Sows immunized with the Flc-LOM-BE^rns^ vaccine have been protected against vertical viral transmission of their fetus when challenged with virulent CSFV at the early, middle, and late period of pregnancy [4]. Lactogenic transfer of maternally derived antibodies (MDAs) from immunized sows is considered an effective and economic way to provide piglets with passive immunity against virus infection before they acquire active immunity via direct vaccination [5,6]. In piglets that have ingested low quantities of colostrum, vaccination induces a strong antigenic stimulus, which is characterized by a normal humoral immune response and resistance to subsequent virus challenge [7,8,9,10,11,12,13]. A number of studies have been conducted to investigate whether pigs that have passive antibodies obtained from colostrum can acquire active immunity after an attenuated live CSF vaccination [7,9]; unfortunately, limited studies were conducted to test the efficacy of passive immunity provided only by colostral antibodies against virulent virus challenge [11,14]. 

Therefore, the aim of this study is to investigate whether pigs that obtained various levels of MDAs from sows immunized with the Flc-LOM-BE^rns^ vaccine are protected against subsequent challenge with virulent CSFV. 

## 2. Results 

### 2.1. MDA Titer and RNA Copy Number 

Ten pigs that obtained MDAs from Flc-LOM-BE^rns^-vaccinated sows were divided into subgroups by their MDA levels: V1 (6 log_2_ MDA), V2 (5 log_2_ MDA), V3 (4 log_2_ MDA), and V4 (3 ≤ log_2_ MDA) (Table 1). MDA levels of 10 pigs according to age of pig were as follows: 42-day-old pigs (6 log_2_ MDA), 40-day-old pigs (5 and 6 log_2_ MDA), 46-day-old pigs (5 log_2_ MDA), 53-day-old pigs (4 and 5 log_2_ MDA), and 60-day-old pigs (3 ≤ log_2_ MDA), respectively (Table 1). For the mock group, five pigs (48-day-old and 55-day-old) that obtained MDAs from unvaccinated sows with 3 ≤ log_2_ MDA (negative) were tested. The MDA levels of all pigs were obtained on the day of challenge and a threshold of 3 ≤ log_2_ was used to determine positive/negative based on serum neutralization titer.

We have used slightly different aged pigs to obtain 4 different MDA levels to test the efficacy of passive immunity (Table 1). After challenge, CSF RNA was detected in only two blood samples (RNA copy number = 1.5 and 2.1 log_10_) from pigs in the V1 subgroup (at 7 days post inoculation, or dpi); however, no CSF RNA was detected in fecal or nasal samples during the 3-week observation period (Table 1). CSF RNA was detected in blood samples from two pigs in the V2 subgroup (at 7 and 10 dpi); viral RNA was detected in blood, nasal, and/or fecal samples from the remaining pigs from 7 to 21 dpi (Table 1). CSF RNA was detected in almost all samples (blood, nasal, and fecal) from pigs in subgroups V3 and V4, and in all samples from mock group pigs, during the observation period (Table 1). 

### 2.2. Clinical Scores, Leucocyte Counts, Body Temperature Measurement, and Mortality 

**Figure 1:**
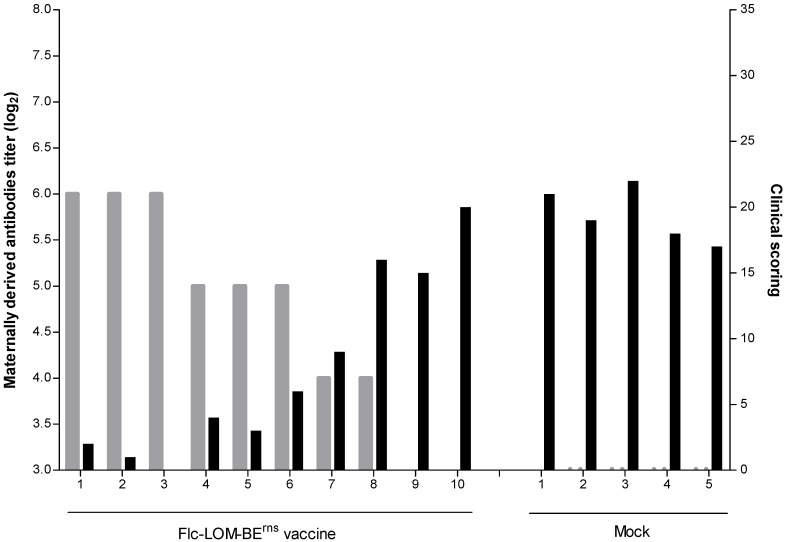
Titers of maternally derive antibodies at the time of challenge and mean clinical signs upon CSFV infection. Pigs acquired passive immunity from sows inoculated with the Flc-LOM-BE^rns^ vaccine. Other pigs received MDA from sows not inoculated with the Flc-LOM-BE^rns^ vaccine (Mock). Maternally derived antibody titers are denoted by light gray bars, and the average clinical scores during the experimental period are marked by black bars.

**Figure 2:**
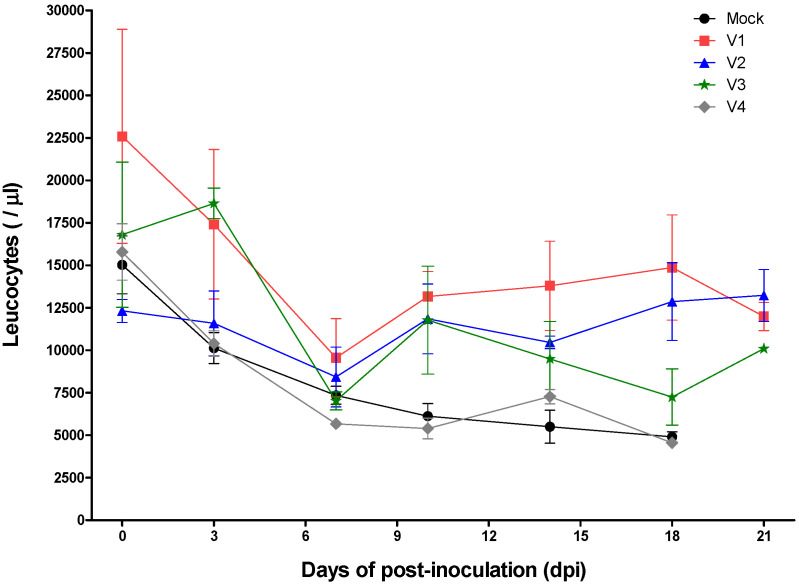
Leucocyte counts post-CSFV infection. Leucocytes from pigs with MDA titers of 6 (V1), 5 (V2), 4 (V3), and 3 ≤ (V4) log_2_ or mock controls are denoted by rectangles (red), triangles (blue), stars (green), diamonds (gray), and circles (black), respectively.

**Figure 3:**
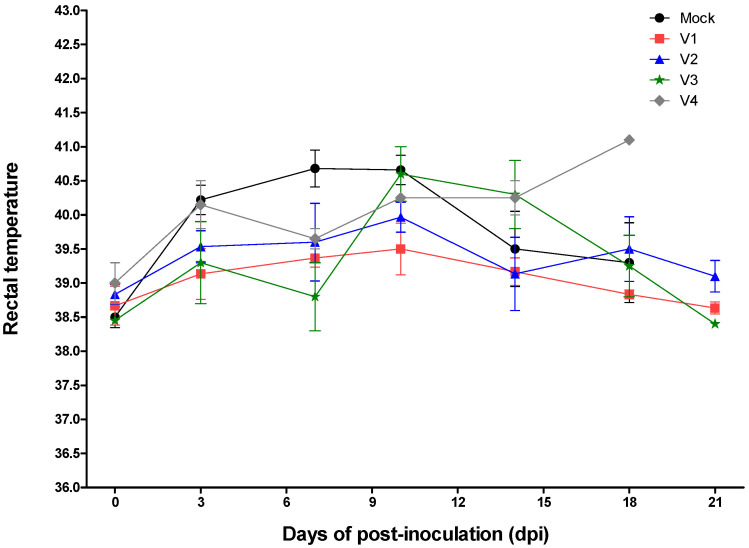
Rectal temperature and maternally derived antibody titers post-CSFV infection. Rectal temperature of pigs with MDA titers of 6 (V1), 5 (V2), 4 (V3), and 3 ≤ (V4) log_2_ and of mock control pigs are marked by rectangles (red), triangles (blue), stars (green), diamonds (gray), and circles (black), respectively.

All groups showed various clinical signs after inoculation with virulent CSFV. The mean clinical scores during the experimental period were as follows: 1 for subgroup V1, 4.3 for subgroup V2, 12.5 for subgroup V3, 17.5 for subgroup V4, and 19.4 for the mock group (Figure 1). The mean number of leucocytes after inoculation with virulent CSFV was as follows: V1 subgroup, no change; V2 subgroup, a small drop only (8433/μL) at 7 dpi; and V3 subgroup, a decline in the leucocyte count at 7 (7025/μL) and 18 (7250/μL) dpi (Figure 2). The mean leucocyte count in the V4 subgroup declined markedly at 7 (5675/μL), 10 (5393/μL), 14 (7270/μL), and 18 (4530/μL) dpi, as did that in mock pigs at 7 (7350/μL), 10 (6120/μL), 14 (5510/μL), and 18 (4940/μL) dpi (Figure 2). The mean rectal temperature of the V1 subgroup changed only very slightly (38.7–39.5 °C) after inoculation with virulent CSFV; however, the rectal temperature in the V2 subgroup was 40.0 °C at 10 dpi, and that in the V3 subgroup was 40.3–40.6 °C at 10–14 dpi (Figure 3). The rectal temperature of pigs in the V4 subgroup and the mock group was high (40.2–41.1 °C at 3–21 dpi and 40.2–40.7 °C at 3–10 dpi, respectively) (Figure 3).

The survival rates of pigs with MDAs from Flc-LOM-BE^rns^ vaccinated and unvaccinated sows after virulent CSFV challenge were 70% and 0%, respectively (Table 1). All pigs in the V2 and V1 subgroup survived when challenged with virulent CSFV; however, the survival rates for those in the V3 and V4 subgroups were 50% (1/2) and 0% (0/2), respectively (Table 1). The three pigs died at 18, 20, and 21 days post-CSFV inoculation (dpi), respectively (Table 1). All five pigs in the mock group died between 12 and 19 dpi (Table 1).

### 2.3. Detection of CSFV RNA in Organs

We performed qRT-PCR to detect CSFV RNA in samples from 11 organs (tonsil, lung, heart, liver, kidney, ileum, cecum, spleen, mesenteric lymph node, inguinal lymph node, and brain) harvested from pigs. No CSFV RNA was detected in any of the organs from the three pigs in the V1 subgroup; however, low RNA copy numbers (2.1–2.9 log_10_) were detected in tonsil, kidney, and spleen from pigs in the V2 subgroup (Table 2). By contrast, CSFV RNA (1.5–4.2 log_10_) was detected in tonsil, lung, heart, kidney, cecum, spleen, mesenteric lymph node, inguinal lymph node, and brain tissue from pigs in the V3 and V4 subgroups. CSFV RNA was also detected (1.4–5.3 log_10_) in tissue samples from various organs from the five pigs in the mock group (Table 1). 

### 2.4. CSFV E^rns^ and BVDV E^rns^ Antibody Titers 

The CSF E^rns^ antibody titer of all 10 pigs with MDAs, at the day of challenge was negative (serological neutralizing antibody titer, or S/N value, 0.67–0.81) according to a CSF E^rns^ ELISA. However, a BVDV E^rns^ ELISA detected BVDV E^rns^ antibodies in 70% (7/10) of the pigs (S/P value, 0.63–0.85) (Table 3). On the day of necropsy, the CSF E^rns^ S/N values of pigs with MDAs (induced by the Flc-LOM-BE^rns^ vaccine) revealed 50% (5/10) of positive antibody (S/N value, 0.32–0.45); however, the BVDV E^rns^ S/P values showed that only 40% (4/10) have positive antibodies (S/P value: 0.60–0.73) (Table 3). The CSF E^rns^ S/N and the BVDV E^rns^ S/P values of pigs from unvaccinated sows (Mock group) showed all negative at 0 dpi and on the day of necropsy after virulent CSFV challenge (Table 3). At the time of necropsy, there was either a very small decrease or no change in MDA serum neutralization antibody titers after inoculation with virulent CSFV (Table 3).

## 3. Discussion

CSFV-specific antibody titers of piglets from immunized sows were declining quickly during the early postnatal weeks. It has been proven that dilution of the MDAs due to the growth of the piglets was the main cause for the decay of the MDAs [15]. In association with the growth of the piglets, the increased catabolism may also affect the decline of the MDAs. The decay of reactivities seemed to be almost linear with clear negative ELISA results at about 10 weeks post farrowing [12]. Depending on the age of piglets, MDAs may have different effects on the development of antibodies by live attenuated CSFV vaccination. The protection rate at the end of the economic life of piglets with colostral antibodies was 50% when they received live attenuated vaccine inoculatation at the age of 7 days, whereas a 100% protection rate was shown when vaccination was given at the age of 2 months [8]. A previous study suggested that vaccination with the Chinese strain is fully effective in piglets that have passive immunity when they are 30–60 days old [7]. Several other reports also indicated that the level of anti-CSFV antibodies titer of 32-fold (5 log_2_) can provide an adequate protection to pigs actively vaccinated with C-strain [9,16], CP7_E2alf [17], or rPRVTJ-delgE/gI-E2 [18]. The piglets with MDAs originating from vaccination of the sows with C-strain were provided full to partial protection against lethal CSFV challenge, depending on the age of pigs [19,20]. MDAs from the sows immunized three times with CP7_E2alf did not protect piglets against CSFV “Koslov” strain challenge, and the piglets showed severe clinical signs [10]. The passive protection provided by MDAs derived from the rAdV-SFV-E2-immunized sow was also observed in the C-strain but not in CP7_E2alf. The serum neutralization titer of the anti-CSFV antibody was 1:49 in the piglets (around the 5-week-old age) born from the sow immunized with 4 x 10^6^ TCID_50_ rAdV-SFV-E2, which can confer a complete protection to piglets [14]. Another study reported that piglets inoculated with virulent CSFV (Shimen strain) at the age of 56 days received MDAs (from sows inoculated with the C-strain vaccine); however, they did not show protection at an MDA titer of 18±3 (serum neutralization antibody titer) [11]. Similar results of no protection were also shown when five pigs with an MDA titer of 16 ± 0 (serum neutralization antibody titer) from sows vaccinated with anti-rAdV-SFV-E2 were challenged with the Shimen strain [11]. 

Interestingly, when the serum neutralization test (SNT) was performed using the virulent strains (YC11WB and ALD) and vaccine strain (LOM) to test the MDA titers of piglets, the MDA titer of the V1-3 subgroups (>3 log_2_) were the same, but the mock group and V4 subgroup (≤3 log_2_) showed slightly different results. Specifically, in the SNT using ALD and YC11WB strains, Pig ID 9 and 10 of piglets of Flc-LOM-BE^rns^ vaccinated sows were <2 log_2_ and 3 log_2_, whereas all piglets of unvaccinated sows were <2 log_2_. In addition, in the SNT by LOM vaccine strain, Pig ID 9 and 10 of piglets of Flc-LOM-BE^rns^ vaccinated sows were all 3 log_2_, and all piglets of unvaccinated sows showed the titer range from <2 to 3 log_2_. 

In the detection of MDAs by the SNT, we have used the SN titer of 3 log_2_ as a threshold and less than 3 log_2_ was considered as negative. This threshold was selected considering the SN titers obtained from control piglets born from unvaccinated sows and the high specificity of serum neutralization assay by immunostaining reading. This decision was made because CSFV vaccination has been mandatory in South Korea for 40 years, and it is possible that some residual levels of MDAs are still detected even in farms that had stopped the vaccination of their sows for a long time.

There are differences among farms and even individual sows with respect to the time that MDA decays. In accordance with recommendations by the South Korea government, Korean pig farmers give pigs one shot (at 55–70 days old) or two shots (at 40 days old and 60 days old) of CSF vaccine (LOM strain) [21]. Anti-CSFV antibodies from slaughtered pigs in South Korea showed a positive rate over 95% each year, which indicates that our CSF vaccination schedules are effective [22]. Our results with the Flc-LOM-BE^rns^ vaccination of sows indicated that when offspring were vaccinated around 45–60 days old, MDA may not provide full protection against virulent CSFV, which supports the vaccination schedules of the LOM CSFV vaccine. Therefore, an active Flc-LOM-BE^rns^ vaccination schedule in pigs also needs to consider these periods of MDA decay time to induce efficient lifelong protection. To select an adequate timeline for an active vaccination schedule for Flc-LOM-BE^rns^ in pigs, further animal experiments may be needed.

Highly virulent, moderately virulent, and avirulent strains of CSFV were tested in standardized animal experiments to confirm their virulence and were used to search for in vitro parameters allowing the differentiation of strains according to their virulence; these observations were also characterized by a newly developed 10 clinical score determination scheme [23]. Clinical scores from 10 parameters were calculated to a maximum of 30 per day [23]. Based on the previously reported clinical sign scoring system, we added the scores for the leucopenia and the dead, and the clinical scores for 12 parameters were summed during the experiment to measure the average. Here, we found that the mean clinical scores of pigs in the V1 and V2 subgroups (6 and 5 log_2_ MDAs) infected with virulent CSFV were 1 and 4.3, respectively. When pigs received a clinical score of less than 3, it was considered as complete protection against virulent CSFV. The mean rectal temperature and leucocyte counts in the V1 subgroup pigs was <40 °C and >8000/μL, respectively; however, those in the V2 subgroup increased their clinical score temporarily (>40 °C and >8000/μL, respectively). No pigs in the V1 subgroup shed the virus via nasal discharges or feces, although CSFV RNA was detected in nasal and fecal swabs from one of the pigs in the V2 subgroup. At the time of necropsy, no CSFV RNA was detected in 11 organs from the V1 subgroup pigs, whereas in two of the three pigs in the V2 subgroup, viral RNA was detected in tonsil, kidney, and spleen samples. Therefore, our data indicated that for a passive immune response induced by MDA, the protective antibody titer should be at least 6 log_2_ or more. 

The different antibodies that function as a DIVA vaccine (Flc-LOM-BE^rns^ strain) in this study were detected in MDAs: all negative CSF E^rns^ S/N values and all positive BVDV E^rns^ S/P values (3 pigs of 5 log_2_ MDAs). When pigs infected with virulent CSF were subjected to necropsy, three pigs with an MDA titer of 6 log_2_ had antibodies specific for both CSF E^rns^ and BVDV E^rns^ at 21 dpi; however, three pigs with an MDA titer of 5 log_2_ showed both positive or negative values at 21 dpi. A previous study showed that pregnant sows inoculated with the Flc-LOM-BE^rns^ vaccine were anti-CSF E^rns^ antibody-negative and anti-BVDV E^rns^ antibody-positive [4]. However, after challenge with virulent CSFV, they were anti-CSF E^rns^ antibody-positive [4]. In other reports, Erns-specific antibodies were induced in some of the piglets challenged at 15 days of post-challenge (dpc) [14] and even suggested to test anti-Erns antibodies at 15 dpc or later for better observation [10]. 

Although a small-scale animal experiment was performed in this study due to the relevance of animal ethical approval, our data showed a correlation with the defense ability and various levels of MDA titers induced by Flc-LOM-BE^rns^ vaccination in sows. Thus, this study can be considered as a first report on the efficiency of MDA against virulent CSFV challenge, and further studies are needed to determine more detailed aspects of the minimum MDA titers to achieve sufficient protection in young pigs.

In conclusion, pigs that received passive immunity via MDAs (≥ 6 log_2_ titer) from sows immunized with the Flc-LOM-BE^rns^ vaccine were protected against virulent CSFV challenge. The Flc-LOM-BE^rns^ vaccine successfully fulfilled the function of differential diagnosis when passive immunity was induced by MDA from immunized sows.

## 4. Materials and Methods

### 4.1. Animal Experiments 

In animal experiments, sows were inoculated with the SuiShot^®^ CSFV Marker-L (Flc-LOM-BE^rns^ strain) vaccine of the CAVAC (Choong Ang Vaccine Laboratories Co., Ltd.). Pigs born from sows that were inoculated with or without the Flc-LOM-BE^rns^ vaccine were divided into two groups and challenged with virulent CSFV at 40, 42, 46, 48, 53, 55, and 60 days of life with different MDA titers (Table 1). Slightly different aged pigs with various levels of MDA were used in the experiment. Ten pigs obtained MDAs from five sows inoculated with the Flc-LOM-BE^rns^ vaccine, and five pigs obtained MDAs from two sows that did not receive the Flc-LOM-BE^rns^ vaccine (Table 1). All pigs were tested for MDA level from serum obtained at the day of challenge. For the challenge of virulent CSFV, YC11WB strain subgenotype 2.1d was injected intranasally (2 ml; 10^5.0^TCID_50_/mL), and the pigs were observed for 3 weeks. At 0, 3, 7, 10, 14, 18, and 21 days post inoculation (dpi), blood samples were collected to investigate levels of MDAs, the CSFV RNA copy number (log_10_), and the number of leucocytes. The clinical score was determined based on 10 parameters (liveliness, body tension, body shape, breathing, walking, skin, eyes, appetite, defecation, and leftovers in the feeding trough), as described previously [23]. The clinical scoring system is 0 (normal), 1 (slightly altered), 2 (showing distinct clinical signs), and 3 (showing severe CSF clinical signs) according to each parameter. Briefly, the clinical score for each of the 3 values for these 10 parameters are as follows: dormant, will not stand up; cramps; emaciated, backbone and ribs visible, head size too big compared to body size; frequency >30/min, breathing through open mouth; massive lameness, unable to walk; black-red discoloration of skin, no sensitivity, large hemorrhages in skin; highly inflamed, purulent secretion, accentuated blood vessels; does not eat at all, shows no interest for food; no feces, mucus in rectum, or watery or bloody diarrhea; trough still full, nothing eaten. The clinical score of 0, 1, or 2 was determined by the degree of the severity. Additionally, we counted the days of leucopenia (<8000/uL), scoring 1 for one day and 3 for the death. The clinical score was measured daily during the experiment period, and only the day the pig died was added with a clinical score of 3. The mean clinical score was measured as the average of the clinical score levels accumulated during the experimental period. Protection was classified as none (total clinical score ≥11), weak (total clinical score 4–10), or strong (total clinical score 0–3). 

After virulent CSFV challenge, pigs were dead within 21 days post inoculation. Necropsy was performed at the time of death, and pigs that survived throughout the observation period were necropsied at 21 days post inoculation. Eleven organs (tonsil, lung, heart, kidney, cecum, spleen, mesenteric lymph node, inguinal lymph node, and brain) were collected by necropsy, and viral copy number were detected by qRT-PCR. 

### 4.2. Serum Neutralization Test and qRT-PCR

To detect CSF-specific serum neutralization antibodies, a neutralization peroxidase-linked antibody (NPLA) assay was performed according to the standards manual of the World Organization for Animal Health [24]. According to the OIE criteria, an initial serum dilution to antibodies examination performed two-fold dilution. Two virulent CSF viruses (ALD strain and YC11WB strain) and one CSF vaccine strain (LOM) using the NPLA reacted 200 TCID_50_/mL per 96-well plate. Less than 3 fold (3 ≤ log_2_) neutralizing antibodies was considered as negative. The VDx^®^ CSFV qRT-PCR (Median Diagnostic Co., Cat No. NS-CSF-31, Cheonchen, South Korea), which employs TaqMan probes, detects the CSFV 5’UTR with high specificity, but it does not detect BVDV or border disease virus, which also belongs to the genus *Pestivirus*. The assay was performed as described previously [25].

### 4.3. CSFV E^rns^ and BVDV BE^rns^ ELISAs 

The CSFV E^rns^ and BVDV E^rns^ ELISAs used to detect specific antibodies were performed as described previously [4]. The VDPro^®^ CSF Erns Ab b-ELISA (MEDIAN Diagnostic Co., Cat No. ES-CSF-05, Korea), a competition ELISA designed to detect the E^rns^ protein, was also used to determine the S/N value (>0.5 negative and 0.5≤ positive). The VDPro^®^ BVDV Erns Ab i-ELISA (MEDIAN Diagnostic Co., Cat No. ES-BVD-01, Korea), which is based on the E2 protein, provided an S/P value (≥0.6 positive and 0.6< negative).

### 4.4. Ethical Approval

The authors confirm that the work complies with the ethical policies of the journal. The work was approved by the Institutional Animal Care and Use Committee of the Animal and Plant Quarantine Agency (APQA) (Approval Number: 2019-481). 

## Figures and Tables

**Table 1:** Viral copy number of pigs with maternally derived antibodies (MDA) from sows vaccinated with the Flc-LOM-BE^rns^ vaccine after challenge. CSFV: classical swine fever virus.

Group ^a^	Sub Group	Pig ID	MDA (log_2_) of Pigs ^b^	Pig Age ^c^	RNA Copy Number (log_10_) from Samples (Blood/Nasal/Feces) on Different Days Post-CSFV Inoculation (dpi)						
					0	3	7	10	14	18	21
Flc-LOM-BE^rns^	V1	1	**6**	42	-/-/-	-/-/-	-/-/-	-/-/-	-/-/-	-/-/-	-/-/-
		2	**6**	42	-/-/-	-/-/-	2.1/-/-	-/-/-	-/-/-	-/-/-	-/-/-
		3	**6**	40	-/-/-	-/-/-	1.5/-/-	-/-/-	-/-/-	-/-/-	-/-/-
	V2	4	**5**	40	-/-/-	-/-/-	1.2/-/-	2.3/-/-	-/-/-	-/-/-	-/-/-
		5	**5**	46	-/-/-	-/-/-	2.3/-/-	1.0/-/-	-/-/-	-/-/-	-/-/-
		6	**5**	46	-/-/-	-/-/-	2.5/-/-	2.8/-/1.5	2.4/1.8/-	2.1/-/-	2.3/1.7/-
	V3	7	**4**	53	-/-/-	-/-/-	2.5/1.8/2.1	3.1/3.7/-	4.3/1.5/2.3	3.5/-/2.5	3.8/2.6/3.1
		8	**4**	53	-/-/-	-/-/-	3.5/2.1/-	4.1/3.2/4.4	5.3/2.5/2.9	4.5/3.3/3.1	D
	V4	9	≤3	60	-/-/-	-/-/-	4.4/2.9/1.6	5.7/3.5/4.2	3.8/2.7/2.6	D	
		10	≤3	60	-/-/-	-/-/-	3.5/-/2.8	5.2/3.3/3.7	4.6/3.4/2.9	3.2/3.6/3.4	D
Unvaccinated	Mock	1	≤3	48	-/-/-	2.6/2.1/1.5	5.1/3.4/3.6	4.4/3.6/3.2	4.5/3.3/3.8	D	
		2	≤3	48	-/-/-	3.4/-/2.7	4.2/2.4/3.8	5.5/3.8/3.1	5.1/3.1/2.4	3.7/4.2/2.5	D
		3	≤3	55	-/-/-	2.9/2.4/-	4.4/3.4/2.2	4.6/3.7/3.6	5.3/2.6/3.4	D	
		4	≤3	55	-/-/-	3.6/2.4/2.7	4.7/2.5/4.2	5.3/2.4/3.7	D		
		5	≤3	55	-/-/-	2.8/3.2/2.5	3.4/3.7/2.5	5.4/4.2/3.5	4.7/4.4/2.8	D	

**^a^** Two groups of pigs (aged 40–60 days) receiving MDAs from sows immunized (or not) with Flc-LOM-BE^rns^ were challenged with virulent CSFV. Then, pigs were divided into subgroups based on their maternally derived antibody levels at the day of challenge: V1 (6 log_2_ MDA), V2 (5 log_2_ MDA), V3 (4 log_2_ MDA), V4 (3 ≤ log_2_ MDA), and Mock (3≤ log_2_ MDA). **^b^** MDA(log_2_) of pigs: MDA of pigs on the day of challenge. Positive and negative MDA of pigs on the day of challenge were marked with bold and normal letters, respectively. Threshold for positive and negative by the serum neutralization antibody test were set to 3 log_2_. **^c^** Pig age: pig age on the day of challenge, D: dead, and -: no detection.

**Table 2:** CSFV viral copy number in different organs from pigs challenged with CSFV, as detected by qRT-PCR.

Group	Sub Group	Pig ID	MDA (log _2_) of Pigs	Pig Age ^a^	RNA Copy Number (log _10_) from Organ Samples on the Necropsy										
					To ^b^	Lu	He	Li	Ki	Il	Ce	Sp	ML	LN	Br
Flc-LOM-BE^rns^	V1	1 2 3	6 6 6	42 42 40	-	-	-	-	-	-	-	-	-	-	-
					-	-	-	-	-	-	-	-	-	-	-
					-	-	-	-	-	-	-	-	-	-	-
	V2	4 5 6	5 5 5	40 46 46	-	-	-	-	-	-	-	-	-	-	-
					2.2	-	-	-	-	-	-	-	-	-	-
					2.5	-	-	-	2.9	-	-	2.1	-	-	-
	V3	7 8	4 4	53 53	3.1	-	-	-	-	-	-	3.4	1.5	1.9	-
					2.7	-	-	-	2.6	-	2.1	-	-	1.5	-
	V4	9 10	≤3 ≤3	60 60	3.6	-	2.7	-	3.5	-	-	-	2.3	2.8	2.4
					2.9	2.8	-	-	4.2	-	-	2.5	4.2	3.7	-
Unvaccinated	Mock	1 2 3 4 5	≤3 ≤3 ≤3 ≤3 ≤3	48 48 55 55 55	3.7	-	-	-	3.4	-	-	4.2	-	4.1	2.9
					4.6	2.8	1.4	-	4.5	-	-	-	-	-	-
					-	-	-	-	2.1	-	3.5	5.3	4.6	3.2	-
					5.3	-	-	-	-	-	-	4.6	3.8	-	3.2
					2.9	4.2	-	-	3.3	-	-	4.1	2.8	3.6	-

^a^Age: pig age on the day of challenge. ^b^To, tonsil; Lu, lung; He, heart; Li, liver; Ki, kidney; Il, ileum; Ce, cecum; Sp, spleen; ML, mesenteric lymph node; LN, inguinal lymph node; Br, brain.

**Table 3:** Comparison of MDA, CSFV E^rns^, and bovine viral diarrhea virus (BVDV) E^rns^ antibody titers before CSFV inoculation and at the time of necropsy after challenge.

Group	Subgroup	Pig ID	Pig Age ^a^	Day of Challenge (0 dpi)			Day of Necropsy			
				MDA ^b^	CSF E^rns^	BVDV E^rns^	SN	CSF E^rns^	BVDV E^rns^	dpi
Flc-LOM-BE^rns^	V1	1	42	6	0.81	**0.76**	6	**0.36**	**0.68**	21
		2	42	6	0.73	**0.85**	7	**0.42**	**0.73**	21
		3	40	6	0.76	**0.77**	6	**0.32**	**0.60**	21
	V2	4	40	5	0.68	**0.83**	6	**0.45**	**0.72**	21
		5	46	5	0.71	**0.75**	5	**0.39**	0.58	21
		6	46	5	0.67	**0.84**	≤3	0.73	0.36	21
	V3	7	53	4	0.79	0.55	≤3	0.81	0.31	21
		8	53	4	0.69	**0.63**	≤3	0.62	0.45	20
	V4	9	60	≤3	0.77	0.40	≤3	0.85	0.18	18
		10	60	≤3	0.72	0.51	≤3	0.79	0.33	21
Unvaccinated	Mock	1	48	≤3	0.89	0.42	≤3	0.76	0.25	17
		2	48	≤3	0.92	0.31	≤3	0.85	0.33	19
		3	55	≤3	0.73	0.18	≤3	0.87	0.42	16
		4	55	≤3	0.90	0.36	≤3	0.82	0.19	12
		5	55	≤3	0.82	0.28	≤3	0.93	0.32	15

^a^Pig age: pig age on the day of challenge. ^b^MDA, maternally derived antibody titer (log_2_); CSF E^rns^, antibody ELISA (S/N value: >0.5 negative and 0.5≤ positive); BVDV E^rns^, antibody ELISA (S/P value: ≥0.6 positive and 0.6< negative); S/N, serological neutralizing antibody titer (log_2_); dpi, day post-CSFV inoculation. Positive samples for the CSFV E^rns^ and BVDV E^rns^ are marked in bold.
